# Genetic and environmental risk factors for stillbirth in Japanese Black cattle

**DOI:** 10.1093/jas/skag248

**Published:** 2026-08-11

**Authors:** Osuke Suzuki, Aisaku Arakawa, Motohide Nishio, Shinya Goto, Mizuho Uematsu, Yosuke Sasaki

**Affiliations:** Agriculture Program, Graduate School of Agriculture, Meiji University, Kawasaki 214-8571, Japan; Institute of Livestock and Grassland Science, National Agriculture and Food Research Organization, Tsukuba 305-0901, Japan; Institute of Livestock and Grassland Science, National Agriculture and Food Research Organization, Tsukuba 305-0901, Japan; Institute of Livestock and Grassland Science, National Agriculture and Food Research Organization, Tsukuba 305-0901, Japan; Production Veterinary Medical Center, Miyazaki Agricultural Mutual Aid Association, Miyazaki 880-0877, Japan; Agriculture Program, Graduate School of Agriculture, Meiji University, Kawasaki 214-8571, Japan

**Keywords:** beef cattle, epidemiology, herd size, modeling, sire

## Abstract

Stillbirth is a critical indicator of production efficiency in the beef industry and leads to substantial economic losses. Although the Japanese Black is a premier breed renowned for superior marbling, research on stillbirth risk factors specific to this breed remains limited. This study aimed to identify epidemiological and genetic factors associated with stillbirth in Japanese Black cattle in order to provide actionable insights for production sites. In total, 94,569 calving records from 24,821 cows across 1,001 farms in the Kyushu region (2009–2018) were analyzed using descriptive statistics, a single-trait threshold animal model with Bayesian inference, and mixed-effect model. The overall average stillbirth rate was 1.91%. Small-scale farms exhibited lower stillbirth rates, with more than 70% reporting no stillbirths, whereas large-scale farms showed higher risk, likely reflecting challenges in individual animal observation. Stillbirth rates varied markedly among sires, with some exceeding the overall average by more than 1%. The posterior mean (±SD) estimates for heritability and repeatability were 0.037 ± 0.024 and 0.119 ± 0.040, respectively. Environmental analyses indicated that first-parity cows (odds ratio: 1.858) and winter calving (odds ratio: 1.390) were associated with significantly increased stillbirth risk (*P* < 0.05). A temporary decline in stillbirth rates was also observed following the 2010 foot-and-mouth disease outbreak, suggesting that reduced herd density and improved care per animal can mitigate risk. Although heritability for stillbirth was low, the relatively high repeatability underscores the importance of managing cows with a prior history of stillbirth. Reducing stillbirth in Japanese Black cattle therefore requires a multifaceted approach, including improved calving management for heifers and winter calvings, use of sire-specific risk information in breeding decisions, and adoption of information and communication technology–based monitoring systems on larger farms. These measures have the potential to reduce economic losses and improve reproductive efficiency.

## Teaser

Our findings indicate that reducing stillbirth rates in Japanese Black cattle should focus primarily on environmental management and informed sire selection.

## Introduction

In the beef industry, the stillbirth rate is a critical indicator of production efficiency. Stillbirths lead to direct income losses due to fetal mortality and increased production costs associated with reproductive delays, resulting in substantial economic damage to farmers (Laster et al. 1973; McDermott et al. 1992; Bellows et al. 2002). The Japanese Black is the most prevalent beef cattle breed in Japan, typically reaching a body length of approximately 140 cm and a body weight of about 750 kg at the time of shipping (Gotoh et al. 2018). This breed is renowned for its superior capacity to produce highly marbled beef and has maintained high genetic purity, with no introduction of foreign genes into the breeding stock for more than a century (Hirooka 2014). Because of high production costs, the market value of a Japanese Black calf is approximately three times that of a Holstein–Friesian calf (Ministry of Agriculture, Forestry and Fisheries of Japan (MAFF) 2025). The MAFF has established strategic goals to improve the reproductive efficiency of beef cattle, with particular emphasis on preventing delivery-related accidents. Accordingly, identifying risk factors associated with stillbirth is of paramount importance (Ministry of Agriculture, Forestry and Fisheries of Japan 2020).

In Japan, previous studies have reported stillbirth rates for beef cows ranging between 2.1% and 2.4% (Ogata et al. 1995; Maeda et al. 2014). By contrast, previous reports indicate higher average stillbirth rates in other breeds: 4.5% in Angus (Hohnholz et al. 2019; Eriksson et al. 2020), 5.6% in Hereford, 5.9% in Charolais (Eriksson et al. 2004), 7.5% in Jersey, 3.5% in Swedish Red (Liedgren et al. 2024), and 5% in Holstein–Friesian cattle (Bicalho et al. 2007; Mahnani et al. 2018). Rates also vary among crossbreeds, including 2.3% for Limousin × Holstein, 4.5% for Simmental × Holstein, 4.4% for Charolais × Holstein (Basiel et al. 2024), and 4.5% for Jersey × Holstein (Liedgren et al. 2024).

Epidemiological research on factors contributing to stillbirth has focused on a range of aspects, including parity (heifers), calving season (winter), and extreme gestation lengths (Uematsu et al. 2013; Sasaki et al. 2014; Mellado et al. 2017). From a physiological perspective, metabolic abnormalities and disease conditions have also been proposed as potential causes (Correa et al. 1993; Mee 2008; Wathes 2012; Mellado et al. 2017). With respect to genetic factors, several studies have reported the heritability of stillbirth; however, most have focused on breeds other than the Japanese Black (Hansen et al. 2004; Hossein-Zadeh 2011; Fonseca et al. 2022). Research specifically addressing Japanese Black cows remains limited, consisting of only one peer-reviewed paper (Oyama et al. 2021) and a master’s thesis (Maeda 2014). Moreover, much of the existing genetic research targets sires for the estimation of breeding values. While farmers under actual production conditions primarily manage dams, making an epidemiological focus on dam-side factors essential for practical insights, evaluating sire effects remains crucial. Specifically, while dam management provides an immediate environmental intervention, informed sire selection offers a complementary, long-term genetic approach to mitigating stillbirth risk. Consequently, the present study used production records to integrate both environmental and genetic factors associated with stillbirth. Our primary objectives were to estimate the heritability and repeatability of stillbirth to inform practical herd management decisions, such as identifying high-risk cows for culling, and to investigate environmental risk factors, including herd size, to highlight the necessity of enhanced monitoring in large-scale operations. Through this comprehensive approach, we aim to provide actionable insights for reducing stillbirth rates in Japanese Black cattle.

## Materials and methods

### Data collection

This study was conducted in a suburban area of the Kyushu region, Japan. Located in the southwestern part of the country (centered at approximately 130.40°E and 33.59°N), Kyushu is characterized by a temperate climate with warm, humid summers and cool winters, and an average annual temperature ranging from 15 to 20°C. It is one of the major beef cattle–producing regions in Japan. In this area, all farms raising Japanese Black cattle register their production records in an electronic database managed by local government–affiliated associations.

The dataset analyzed in this study comprised 95,294 calving records from 24,821 cows across 1,001 farms, collected between 2009 and 2018. The information included parity, calving date, calving outcome (stillbirth or normal birth), cow (dam) ID, service sire ID (the sire of the calf), dam-sire ID (the sire of the cow), and dam-grandsire ID (the maternal grandsire of the cow). Because the raw data consisted of individual records for each calf, the following two procedures were applied to address multiple births (e.g., twins): first, if all calves in a multiple birth had the same outcome (all normal or all stillbirths), one record was randomly retained and the others were excluded to treat the event as a single delivery; and second, if a multiple birth resulted in both stillbirth and normal birth (heterogeneous outcome), the delivery was classified as a single stillbirth, and the record for the live birth was deleted. After these adjustments, a total of 94,569 calving records for 24,821 cows across 1,001 farms were retained for subsequent analyses.

### Data description

Descriptive statistics were used to characterize the dataset and the occurrence of stillbirth. Stillbirth rates were calculated per farm to provide an overview of the regional situation. In this study, stillbirth was defined as fetal death occurring 240 days or later after conception (Uematsu et al. 2013; Sasaki et al. 2014). A chi-square test was used to compare stillbirth rate categories across different herd sizes (*P* < 0.05). The stillbirth rate per farm was classified into four categories: 0% (no stillbirths), >0% to 2%, >2% to 4%, and >4%. Herd size was categorized into three groups: small (1–10 cows; 471 farms), medium (11–50 cows; 368 farms), and large (≥51 cows; 162 farms). In addition, stillbirth rates were calculated per sire, focusing on major sires used for breeding with at least 100 artificial insemination (AI) records.

### Statistical analysis

Two main analyses were performed to identify factors associated with stillbirth. First, genetic parameters for stillbirth were estimated using a single-trait threshold animal model evaluating the cow (dam). The model is defined as:


$$y=X\beta +Z_{s}s+\mathrm{Za}+\mathrm{Wpe}+e$$

where **y** is the vector of observations (1 for stillbirth, 0 for normal birth); β is the vector of fixed effects (parity, calving year, calving season, and farm); **s**, **a**, **pe**, and **e** are vectors of random effects for sire, additive genetic effect (breeding value) of the dam, permanent environmental effect of the dam, and residual, respectively; and **X**, $Z_{s}$**, Z**, and **W** are the corresponding design matrices. Parity was categorized into 10 groups (1–9 and ≥10). Seasons were classified as winter (December–February), spring (March–May), summer (June–August), and autumn (September–November). The additive genetic effects were assumed to follow $a\;∼\;N(0,A\sigma_{a}^{2})$, where $\sigma_{a}^{2}$ is the additive genetic variance and A is the pedigree-based relationship matrix. Similarly, we assumed $s\;∼\;N(0,\;I\sigma_{s}^{2})$, $\mathrm{pe}\;∼\;N(0,\;I\sigma_{pe}^{2})$, and $e\;∼\;N(0,\;I\sigma_{e}^{2})$, where $\sigma_{s}^{2}$, $\sigma_{pe}^{2}$ and $\sigma_{e}^{2}$ are the sire, permanent environment, and residual variance, respectively, and I is an identity matrix. The A matrix was constructed for the cows (dams) by tracing back their pedigrees using the dam-sire and dam-grandsire information. Animals with missing ancestors were assigned unknown parent IDs, ensuring the proper construction of the relationship matrix across the 25,034 animals. Variance components were estimated using Bayesian inference with the Gibbs sampling method implemented in Gibbsf90+ software (Misztal et al. 2002, 2018). The chain length was 1,000,000 iterations, with the first 500,000 discarded as burn-in. Every 100th sample was retained (thinning), yielding a final effective sample size of 5,000. Postgibbsf90 was used to calculate posterior means, posterior standard deviations (SDs), and heritability. Convergence was assessed by visual inspection of trace plots.

Second, environmental factors associated with stillbirth were identified using logistic regression models implemented in SAS software (ver. 9.4; SAS Institute Inc., Cary, NC, USA). The dependent variable was the occurrence of stillbirth (1 or 0), and the independent variables were parity, calving season, and calving year. Farm and sire were included as random effects. Odds ratios (ORs) and 95% confidence intervals (95% CIs) were estimated for each significant factor.

## Results

**Figure 1:**
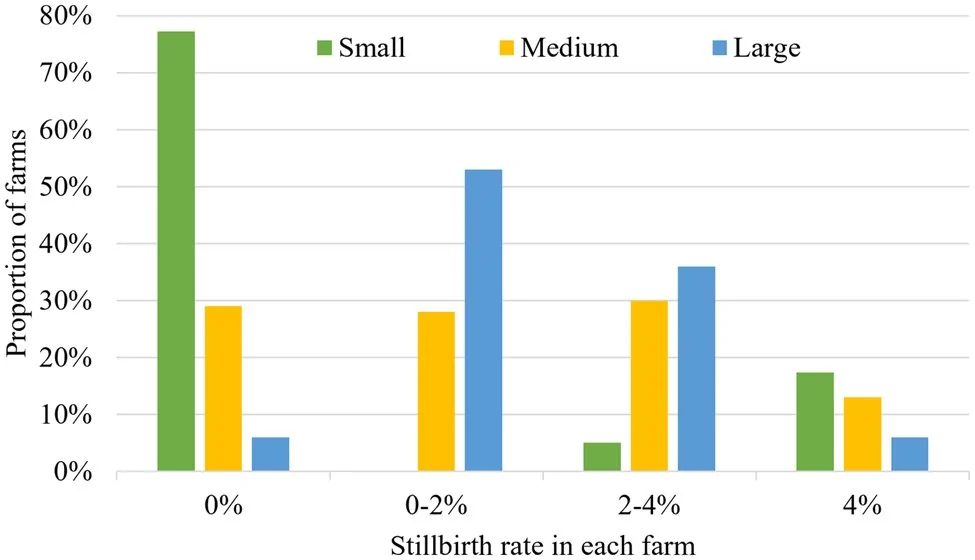
Relative frequencies of stillbirth rate categories across herd size groups.

Between 2009 and 2018, the overall average stillbirth rate on Japanese Black cattle farms was 1.91%, and the mean parity was 4.78. With respect to the distribution of herds by stillbirth rate, approximately 48%, 19%, 20%, and 13% of herds fell into the 0%, >0%–2%, >2%–4%, and >4% categories, respectively. Figure 1 illustrates the distribution of stillbirth rates by herd size and shows a significant difference among herd size categories (*P* < 0.05). Notably, more than 70% of small herds reported no stillbirths.

**Figure 2:**
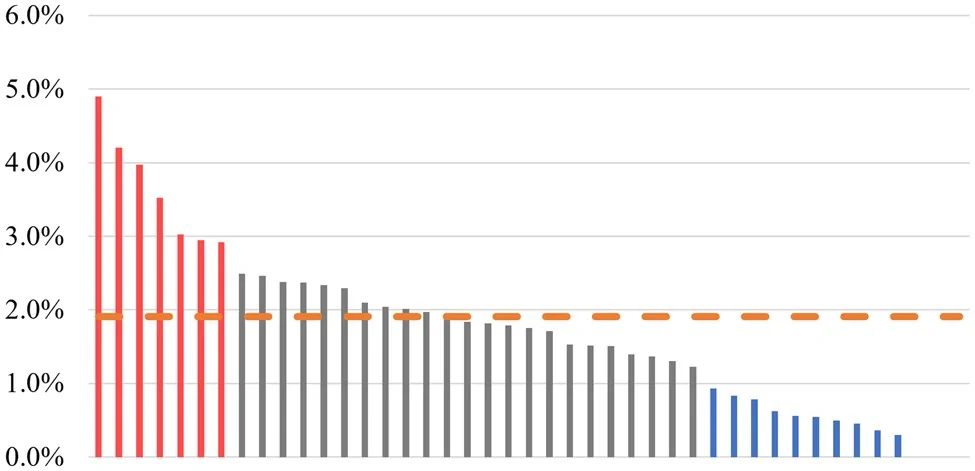
Stillbirth rates for individual sires. The sires shown include 43 sires with 100 or more artificial inseminations during the data collection period. The dotted line indicates the average stillbirth rate.

Figure 2 presents stillbirth rates for individual sires in descending order. This analysis included 43 major sires with at least 100 AI records during the study period. Relative to the overall average stillbirth rate (1.91%), 7 bulls (16%) showed stillbirth rates more than 1% higher, whereas 13 bulls (30%) exhibited rates more than 1% lower.

Table 1 summarizes the estimated variance components and heritability for stillbirth. The estimated variance components ranged from 0.030 ± 0.022 for the sire effect to 0.147 ± 0.055 for the permanent environmental effect. The posterior mean (±SD) estimates for heritability and repeatability were 0.037 ± 0.024 and 0.119 ± 0.040, respectively. Furthermore, to evaluate the genetic liability transmitted to the daughters, the EBVs for all 214 sires of the cows (dam-sires) included in the relationship matrix were extracted from the additive genetic effects in the animal model. The sire EBVs showed a mean of 0.000276 with an SD of 0.003560. The median EBV was 0.000315, with an interquartile range from −0.002292 to 0.002886, and an overall range from −0.009200 to 0.010557. This distribution illustrates the extent of genetic variation available for identifying sires capable of improving calving outcomes in their future daughters.

**Table 1:** Posterior means, heritability, and repetition rates for each variable.

	Variable			Heritability	Repeatability
	*s*	*a*	*pe*		
**Posterior mean**	0.030	0.046	0.147	0.037	0.119
**Posterior SD**	0.022	0.030	0.055	0.024	0.040

SD, standard deviation.

Associations between environmental factors and stillbirth are shown in Table 2. Compared with cows in their second parity (reference), the OR for stillbirth in first-parity cows was significantly higher at 1.858 (95% CI: 1.567–2.203, *P* < 0.05). With respect to calving season, and using summer as the reference, the ORs for winter and autumn were significantly elevated at 1.390 (95% CI: 1.219–1.584) and 1.157 (95% CI: 1.012–1.324), respectively (*P* < 0.05). Compared with calving in 2018 (reference), the ORs for calving years between 2013 and 2017 were significantly lower, ranging from 0.362 to 0.781 (*P* < 0.05).

**Table 2:** Comparison of stillbirth rate by parity, calving season, and calving year.

	*N*	SB, **%**		Estimate		SEM		*P*-value		Odds ratio	95**%** CI
**Parity**											
**1**	13,948	3.04%		0.6196		0.0869		<0.0001		1.858	1.567–2.203
**2**	12,571	1.81%		Ref.							
**3**	11,671	1.40%		−0.2620		0.1040		0.0117		0.770	0.628–0.943
**4**	10,909	1.73%		−0.0601		0.1001		0.5483		0.942	0.774–1.146
**5**	9,996	1.57%		−0.1839		0.1056		0.0816		0.832	0.676–1.023
**6**	9,015	1.45%		−0.2743		0.1116		0.014		0.760	0.611–0.946
**7**	7,760	1.95%		0.0136		0.1072		0.8987		1.014	0.822–1.251
**8**	6,403	1.45%		−0.2922		0.1252		0.0196		0.747	0.584–0.954
**9**	4,869	2.20%		0.1146		0.1200		0.3396		1.121	0.886–1.419
**≥10**	7,427	2.18%		0.0894		0.1058		0.398		1.094	0.889–1.346
**Calving season**											
**Winter**	21,806	2.27%		0.3291		0.0668		<.0001		1.390	1.219–1.584
**Spring**	22,763	1.89%		0.1293		0.0692		0.0617		1.138	0.994–1.303
**Summer**	27,069	1.64%		Ref.							
**Autumn**	22,931	1.89%		0.1460		0.0686		0.0333		1.157	1.012–1.324
**Calving year**											
**2009**	10,661	2.79%		−0.0380		0.1234		1.0000		0.963	0.756–1.226
**2010**	9,884	2.57%		−0.0624		0.1233		1.0000		0.939	0.738–1.196
**2011**	9,014	2.15%		−0.1624		0.1208		1.0000		0.850	0.671–1.077
**2012**	9,303	2.16%		−0.0949		0.1181		1.0000		0.909	0.721–1.146
**2013**	9,011	1.05%		−0.7832		0.1378		<.0001		0.457	0.349–0.599
**2014**	8,859	0.82%		−1.0163		0.1471		<.0001		0.362	0.271–0.483
**2015**	8,863	1.40%		−0.4973		0.1232		0.0024		0.608	0.478–0.774
**2016**	9,405	1.78%		−0.2475		0.1087		1.0000		0.781	0.631–0.966
**2017**	9,704	1.67%		−0.3465		0.1060		0.0487		0.707	0.574–0.870
**2018**	9,865	2.40%		Ref.							

SB, stillbirth; SEM, standard error of the mean; CI, confidence interval.

## Discussion

This study aimed to provide insights into reducing stillbirth rates by conducting descriptive analyses, estimating heritability, and examining environmental factors across 1,001 Japanese Black cattle farms in the Kyushu region. The overall average stillbirth rate was 1.91%, which is lower than that reported in previous studies on Japanese Black cows and substantially lower than rates observed in other beef and dairy breeds (Eriksson et al. 2004; Liedgren et al. 2024). Possible contributing factors include the relatively low birth weight of Japanese Black calves compared with other breeds (Ogata et al. 1995; Dhakal et al. 2013), as well as the high level of calving assistance and specialized management practices commonly adopted by Japanese farmers.

Although the estimated heritability of stillbirth was low (0.037 ± 0.024), continuous selective breeding over the long term can contribute to a gradual reduction in stillbirth incidence. Nevertheless, the limitations of individual cow selection at the farm level should be acknowledged. In Japanese commercial beef production, economic factors, specifically the high market value of Japanese Black calves and the priority of retaining as many breeding heifers as possible to maintain herd size, often override strict culling policies based solely on a history of stillbirth. Previous studies have reported heritability estimates ranging from 0.002 to 0.100 (Meyer et al. 2001; Hansen et al. 2004; Oyama et al. 2021). In addition, the repeatability estimated in this study was higher than that reported in earlier work (ČíTek et al. 2011; Hossein-Zadeh 2011). Given that cows with a prior stillbirth are more likely to experience recurrence, producers should prioritize such animals for enhanced calving management or consider them as primary candidates for culling during herd replacement.

The present results also demonstrated substantial variation in stillbirth risk among sires, with some exceeding the overall average by more than 1%. This finding suggests that sire-specific stillbirth risk should be incorporated into breeding strategies. For example, using semen from low-risk sires for first-calf heifers or for cows with a history of stillbirth may be an effective approach to mitigating risk (Misaka et al. 2022; Constantin and Mihaela 2023).

The lower incidence of stillbirth observed on small-scale farms suggests that management practices associated with herd size influence outcomes. On small farms, frequent observation of pregnant cows and more intensive nighttime monitoring may facilitate earlier detection of abnormal calving. By contrast, as herd size increases, individual observation becomes more difficult, potentially increasing risk. Accordingly, large-scale farms are encouraged to adopt information and communication technology (ICT) to improve monitoring efficiency and to employ staff with specialized skills (MAFF 2018). Other herd-size–related factors that may affect stillbirth include variation in employee skill levels, increased infectious disease risk associated with personnel turnover, challenges in individualized nutritional management, and environmental stressors (Saito et al. 2009; Henker et al. 2020; Antanaitis et al. 2022). Notably, although small farms had a lower overall stillbirth rate, they accounted for the highest proportion of herds with rates exceeding 4%. This likely reflects a mathematical effect, whereby a single stillbirth in a very small herd results in a disproportionately high percentage.

With respect to environmental factors, significantly higher stillbirth rates were observed during winter and among first-parity cows. These findings are consistent with previous reports implicating factors such as incomplete maternal development and fetal malpresentation (Vernooy et al. 2007; Uematsu et al. 2013; Mee 2020). The low heritability estimate further emphasizes that environmental management represents the most immediate means of reducing stillbirth. Interestingly, stillbirth rates were significantly lower during the period from 2013 to 2017. This interval corresponds to the recovery phase following the 2010 foot-and-mouth disease outbreak (MAFF 2010). Mandatory culling during the outbreak resulted in a temporary reduction in herd size, and the subsequent gradual restocking led to lower herd densities for several years. This likely allowed for improved individual animal care per worker, resembling conditions on small-scale farms and contributing to improved stillbirth outcomes (Misaka et al. 2024).

A methodological limitation of the present study concerns the handling of multiple births. Because our genetic analysis utilized a dam-centric model, retaining multiple calf records for a single calving event caused the mathematical model to misinterpret them as multiple, separate deliveries within the exact same parity. Instead of entirely excluding multiple-birth events, a single record was retained because maximizing the number of repeated calving records per cow is crucial for accurately estimating the permanent environmental effect and repeatability. This approach preserved the continuity of the dam’s reproductive history while satisfying the structural requirements of the modeling software. Although incorporating a ‘birth type’ (single vs. twin) effect into the model would ideally allow for the retention of all phenotypic data, the natural incidence of twinning in Japanese Black cattle is extremely low. Therefore, the exclusion of these specific records is expected to have a negligible impact on the overall estimates of genetic parameters and environmental risk factors.

In conclusion, this study clarified the current status and major risk factors associated with stillbirth in Japanese Black cattle in the Kyushu region. Although the overall stillbirth rate (1.91%) was lower than that reported for other breeds, substantial variability was evident depending on herd size, sire selection, and individual cow history. The relatively low heritability (0.037) indicates that environmental and management interventions remain the most effective short-term strategies for reducing stillbirth. In particular, intensive monitoring of first-calf heifers and heightened attention during the winter season are critical. Moreover, the relatively high repeatability of stillbirth suggests that culling cows with a history of stillbirth, or avoiding their offspring as replacement heifers, is a practical and effective option for producers. Wider adoption of ICT to enhance individual animal monitoring on large-scale farms, together with the use of sire-specific stillbirth risk information in breeding programs, is expected to further improve production efficiency and animal welfare in the Japanese beef industry.

## Data Availability

None of the data were deposited in an official repository.

## Abbreviations

AI: artificial insemination
MAFF: Ministry of Agriculture, Forestry and Fisheries of Japan
